# The Effects of Intra-membrane Viscosity on Lipid Membrane Morphology: Complete Analytical Solution

**DOI:** 10.1038/s41598-018-31251-6

**Published:** 2018-08-27

**Authors:** Mahdi Zeidi, Chun Il Kim

**Affiliations:** Department of Mechanical Engineering, University of Alberta, Edmonton, Alberta T6G 2G8 Canada

## Abstract

We present a linear theory of lipid membranes which accommodates the effects of intra-membrane viscosity into the model of deformations. Within the Monge parameterization, a linearized version of the shape equation describing membrane morphology is derived. Admissible boundary conditions are taken from the existing non-linear model but reformulated and adopted to the present framework. We obtain a complete analytical expression illustrating the deformations of lipid membrane subjected to the influences of intra-membrane viscosity. The result predicts wrinkle phenomena in the event of membrane-substrate interactions. Finally, we mention that the obtained solutions reduce to those from the classical shape equation when the viscosity effects are removed.

## Introduction

Lipid membranes are composed of transversely oriented lipid molecules (phospholipids) containing hydrophilic head groups and hydrophobic tails. These phospholipids arrange themselves into a two-layered sheet (a bilayer) with opposing orientations that effectively shield the tail groups from the surrounding aqueous solution. It was found by Evert Gorter and F. Grendel^1^ that a lipid bilayer constitutes cell membrane and later, in 1959, David Robertson^2^ justified that the bilayer structure is characteristic of all biological membranes (biomembranes). They are quite fragile and negligibly thin (typically 512610 nm) but represent a critically important interface within biological cells mediating interactions between cells and their surrounding environment through cellular processes such as fission, fusion and budding^3,4^. These cellular functions are facilitated by the membranes morphological transition which is also dependent on the membrane forces and surrounding viscous flows^5,6^. Therefore, the study of the morphological aspects of membranes is crucial to the understanding of a wide range of essential cellular functions.

Due to the delicate and complex nature of lipid membranes, the study of the various mechanical responses of lipid bilayers can be, most often, practiced with the use of an artificial ‘model’. This also includes the development of continuum-based models in the description of the behavior of lipid bilayers, typically based on the Cosserat theory of elastic surfaces (see^7^, and the reference therein). Within this context, the work in^8^ reveals that intra-membrane viscosity has considerable effects on the deformation of lipid membranes. The authors in^9^ develop the non-linear models of membranes which incorporates intra-membrane viscosity through the adjustment of the equilibrium and boundary conditions based on the conventional theory of elastic surfaces. There the authors further corroborate the relations between lipid flow and membrane shape via the numerical analysis of the resulting coupled PDEs. However, the analysis presented in^9^ is limited to the case of a rectangular portion of lipid membranes whereas membrane morphology is oftentimes affected by external forces such as interactions between membranes and substrates. In addition, it seems necessary to develop a linear theory in order to facilitate further researches on the related subjects.

In the present work, we reformulate the non-linear governing equations of membranes directly from the membrane free-energy density function within the frame work of tensor analysis of surfaces. A compatible linear theory is then developed by employing the principles of superposed incremental deformations. More importantly, we obtained complete analytical solutions which describes the deformations of lipid membranes subjected to intra-membrane viscous flow. Emphasis is placed on the assimilation of the complex nature of boundary forces and the film/substrate interaction conditions, while maintaining the rigor and sufficient generality in the derivation of linearized shape equations and boundary conditions. The obtained linear model predicts the straining effects when a rectangular portion of membranes is subjected to intra-membrane viscous flow. The result is also aligned with the numerical study conducted under the compatible settings^9^. In particular, we find that the viscous flows give rise to wrinkle phenomena when the membrane makes contact with a circular substrate. Quantitative comparison is made by assimilating the experimental results reported in^10^ where we find that the number of wrinkles is sensitive to the thickness of membranes and the radius of interacting substrates. A phenomenologically compatible result is also reported in the work of^11,12^. We believe that the results may provide an important breakthrough in the study of relevant problems such as the effects of wrinkles on the vesicle fusion/diffusion processes^13^ and a wrinkle-caused disease of human eyes (e.g. a macular pucker/epiretinal membrane)^14^. Lastly, we note that our solution also accommodates the scenarios presented in^15,16^ in the limit of vanishing viscous flow.

Throughout the paper, we make use of a number of well-established symbols and conventions. Thus, unless otherwise stated, Greek indices take the values 1 and 2. Einstein summation is applied for the repeated indices.

## Prerequisite

The theoretical formulation of a lipid membrane which accounts for the effects of intra-membrane viscosity is presented in^9^. There the authors obtain the constitutive relation from the theory of an elastic surface via the relation *W* = *ρF* and later by substituting viscosity terms in the resulting formulae. In this section, we reformulate the results directly from the membrane free-energy density *W* = *W*(*H*, *K*, *ρ*) for the sake of consistency and completeness.

### Inviscid lipid membranes

The equilibrium state of a purely elastic surface, subjected to normal pressure *p*, is given by^17^

1 $${{\bf{T}}}_{;\alpha }^{\alpha }+p{\bf{n}}=0,$$

where **T**^*α*^ and **n** are the *stress vectors* and the local surface unit normal, respectively. The semi-colon denotes the surface covariant differentiation associated with the Levi-Civita contraction of the surface metric *a*_*αβ*_ = **a**_*α*_ · **a**_*β*_, where **a**_*α*_ = **r**_,*α*_(*θ*^*α*^, *t*) = ∂**r/**∂*θ*^*α*^. For instance, **a**_*α*;*β*_ is defined by^18^

2 $${{\bf{a}}}_{\alpha ;\beta }={{\bf{a}}}_{\alpha ,\beta }-{{\rm{\Gamma }}}_{\alpha \beta }^{\lambda }{{\bf{a}}}_{\lambda }.$$

Here ${{\rm{\Gamma }}}_{\alpha \beta }^{\lambda }={{\bf{a}}}_{\alpha ,\beta }\cdot {{\bf{a}}}^{\lambda }$ are the Christoffel symbols induced by the local surface coordinate ${\bf{n}}({\theta }^{\alpha })=\tfrac{1}{2}{\varepsilon }^{\alpha \beta }{{\bf{a}}}_{\alpha }\times {{\bf{a}}}_{\beta }$ such that **n** is a unit-vector field and **a**_*α*_ and **a**_*β*_ are the tangent vectors on the deformed surface *ω*. ${\varepsilon }^{\alpha \beta }={e}^{\alpha \beta }/\sqrt{a}$ refers to the permutation tensor with *a* = det(*a*_*αβ*_). Thus, for example, we evaluate *e*^*αβ*^ as *e*^11^ = *e*^22^ = 0, *e*^12^ = −*e*^21^ = 1. The matrix *a*_*αβ*_ of the surface metric is a positive-definite, with *a* > 0, leading to the existence of dual metric *a*^*αβ*^ which is the inverse of the surface metric (i.e. *a*^*αβ*^ = (*a*_*αβ*_)^−1^). Hence the to the dual basis is given as **a**^*α*^ = *a*^*αβ*^**a**_*β*_. The energy induced on the membranes’ deformations can be expressed via the two primary parameters: the coefficient of the first fundamental form *a*_*αβ*_ (the surface metric); and the second fundamental form *b*_*αβ*_ (the curvature) defined by *b*_*αβ*_ = **n** · **a**_*α*,*β*_. For example, in the case of the surface with energy density *W* = *W*(*a*_*αβ*_, *b*_*αβ*_), **T**^*α*^ take the following compact form^17^

3 $${{\bf{T}}}^{\alpha }=({\sigma }^{\beta \alpha }+{b}_{\mu }^{\beta }{M}^{\mu \alpha }){{\bf{a}}}_{\beta }-{M}_{;\beta }^{\alpha \beta }{\bf{n}},$$

where

4 $${\sigma }^{\beta \alpha }=\rho (\tfrac{\partial \tfrac{1}{\rho }(W-\gamma )}{\partial {a}_{\alpha \beta }}+\tfrac{\partial \tfrac{1}{\rho }(W-\gamma )}{\partial {a}_{\beta \alpha }})\,{\rm{and}}\,{M}^{\beta \alpha }=\frac{\rho }{2}(\tfrac{\partial \tfrac{1}{\rho }(W-\gamma )}{\partial {b}_{\alpha \beta }}+\tfrac{\partial \tfrac{1}{\rho }(W-\gamma )}{\partial {b}_{\beta \alpha }}).$$

In the above, *ρ* and *γ* are the surface mass density and the constitutively-indeterminate Lagrange-multiplier field, respectively. For lipid membranes whose free-energy density is expressed by the mean and Gaussian curvatures through *a*_*αβ*_ and *b*_*αβ*_ (i.e. *W* = *W*(*H*, *K*, *ρ*;*a*_*αβ*_, *b*_*αβ*_)), the expressions of *σ*^*βα*^ and *M*^*βα*^ can be obtained by using chain rules $({\rm{e}}.\,{\rm{g}}.\,\frac{\partial W}{\partial {a}_{\alpha \beta }}=\frac{\partial W}{\partial \rho }\frac{\partial \rho }{\partial {a}_{\alpha \beta }}+\frac{\partial W}{\partial H}\frac{\partial H}{\partial {a}_{\alpha \beta }}+\frac{\partial W}{\partial K}\frac{\partial K}{\partial {a}_{\alpha \beta }})$. To see this, we evaluate

5 $$\begin{array}{rcl}\rho \frac{\partial \frac{1}{\rho }(W-\gamma )}{\partial {a}_{\alpha \beta }} & = & \rho [\,-{\rho }^{-2}\frac{\partial \rho }{\partial {a}_{\alpha \beta }}W+\frac{1}{\rho }({W}_{\rho }\frac{\partial \rho }{\partial {a}_{\alpha \beta }}+{W}_{H}\frac{\partial H}{\partial {a}_{\alpha \beta }}+{W}_{K}\frac{\partial K}{\partial {a}_{\alpha \beta }})\\  &  & +\,{\rho }^{-2}\frac{\partial \rho }{\partial {a}_{\alpha \beta }}\gamma -\frac{1}{\rho }\frac{\partial \gamma }{\partial \rho }\frac{\partial \rho }{\partial {a}_{\alpha \beta }}],\end{array}$$

where the expressions of *H* and *K* are given explicitly as^18^

6 $$H=\tfrac{1}{2}{a}^{\alpha \beta }{b}_{\alpha \beta }\,{\rm{and}}\,K=\tfrac{1}{2}{\varepsilon }^{\alpha \beta }{\varepsilon }^{\lambda \mu }{b}_{\alpha \lambda }{b}_{\beta \mu }.$$

The derivatives of *ρ*, *H* and *K* with respect to *a*_*αβ*_ can be evaluated as^17^

7 $$\frac{\partial \rho }{\partial {a}_{\alpha \beta }}=-\,\frac{\rho }{2}{a}^{\alpha \beta },\,\frac{\partial H}{\partial {a}_{\alpha \beta }}=-\,\frac{1}{2}{b}^{\alpha \beta }\,{\rm{and}}\,\frac{\partial K}{\partial {a}_{\alpha \beta }}=-\,K{a}^{\alpha \beta },$$

Accordingly, Eq. (5) reduces to

8 $$\begin{array}{rcl}\rho \frac{\partial \frac{1}{\rho }(W-\gamma )}{\partial {a}_{\alpha \beta }} & = & \rho [\frac{1}{2\rho }{a}^{\alpha \beta }W-\frac{1}{2}{a}^{\alpha \beta }{W}_{\rho }-\frac{1}{2\rho }{b}^{\alpha \beta }{W}_{H}\\  &  & -\,\frac{1}{\rho }{W}_{K}K{a}^{\alpha \beta }-\frac{1}{2\rho }{a}^{\alpha \beta }\gamma +{\gamma }_{\rho }\frac{1}{2}{a}^{\alpha \beta }].\end{array}$$

Now using *F*_*ρ*_ = (*W*/*ρ* − *γ*/*ρ*)_,*ρ*_ $({\rm{i}}.\,{\rm{e}}.\,{W}_{\rho }=\rho {F}_{\rho }+\frac{W}{\rho }+{\gamma }_{\rho }-\frac{\gamma }{\rho })$, we obtain

9 $$\begin{array}{rcl}\rho \frac{\partial \frac{1}{\rho }(W-\gamma )}{\partial {a}_{\alpha \beta }} & = & [\,-\,\frac{1}{2}{b}^{\alpha \beta }{W}_{H}-{W}_{K}K{a}^{\alpha \beta }-\frac{1}{2}{a}^{\alpha \beta }{\rho }^{2}{F}_{\rho }]\\  & = & -\,\frac{1}{2}\gamma {a}^{\alpha \beta }-({W}_{H}H+{W}_{K}K){a}^{\alpha \beta }+\frac{1}{2}{W}_{H}{\tilde{b}}^{\alpha \beta },\end{array}$$

where *F*_*ρ*_ is defined via the relation *ρ*^2^*F*_*ρ*_ = *γ*^17^ and ${b}^{\alpha \beta }=2H{a}^{\alpha \beta }-{\tilde{b}}^{\alpha \beta }$. Thus,

10 $${\sigma }^{\beta \alpha }=(\lambda +W){a}^{\alpha \beta }-(2{W}_{H}H+2{W}_{K}K){a}^{\alpha \beta }+{W}_{H}{\tilde{b}}^{\alpha \beta },$$

and

11 $$\lambda =-\,(\gamma +W).$$

Similarly, by using

12 $$\frac{\partial \rho }{\partial {b}_{\alpha \beta }}=0,\,\frac{\partial H}{\partial {b}_{\alpha \beta }}=\frac{1}{2}{a}^{\alpha \beta }\,{\rm{and}}\,\frac{\partial K}{\partial {b}_{\alpha \beta }}={\tilde{b}}^{\alpha \beta },$$

Eq. (4)_2_ yields

13 $${M}^{\beta \alpha }=\frac{1}{2}{W}_{H}{a}^{\alpha \beta }+{W}_{K}{\tilde{b}}^{\alpha \beta }.$$

Consequently, by substituting Eq. (3) into (1), we have

14 $${({\sigma }^{\beta \alpha }+{b}_{\mu }^{\beta }{M}^{\mu \alpha })}_{;\alpha }{{\bf{a}}}_{\beta }+({\sigma }^{\beta \alpha }+{b}_{\mu }^{\beta }{M}^{\mu \alpha }){{\bf{a}}}_{\beta ;\alpha }-{({M}_{;\beta }^{\alpha \beta })}_{;\alpha }{\bf{n}}-{M}_{;\beta }^{\alpha \beta }{{\bf{n}}}_{,\alpha }+p{\bf{n}}={\bf{0}}.$$

Applying Euclidean dot products in normal **n** and tangential **a**_*α*_ directions and invoking Gauss and Weingarten equations^18^ **a**_*β*;*α*_ = *b*_*βα*_**n** and **n**_,*α*_ = −*b*_*αβ*_**a**^*β*^, Eq. (14) becomes

15 $$({\sigma }^{\beta \alpha }+{b}_{\mu }^{\beta }{M}^{\mu \alpha }){b}_{\beta \alpha }-{({M}_{;\beta }^{\alpha \beta })}_{;\alpha }+p={\bf{0}},$$

and

16 $${({\sigma }^{\beta \alpha }+{b}_{\mu }^{\beta }{M}^{\mu \alpha })}_{;\alpha }+{M}_{;\mu }^{\alpha \mu }{b}_{\alpha }^{\beta }={\bf{0}}.$$

Eq. (15) is often referred to as a membrane shape equation when used in conjunction with Helfrich potential^19^.

### Viscous lipid membranes

Viscous stress arises due to the straining effects of the fluid and can be accommodated by the time derivative of the evolving surface metric^20^. In a typical environment, where lipid membranes are subjected to morphological transitions, the reference velocity of the system is low and therefore the corresponding Reynolds numbers are sufficiently small^21,22^. Further, it is widely accepted that lipid membranes are relatively stiff against areal dilation in comparison with bending or shear motions^23,24^. Thus, in the forthcoming derivations, we adopt the assumption of incompressible fluid and thereby find the expression of the corresponding stress as

17 $${\sigma }^{\alpha \beta }=-\,\gamma {a}^{\alpha \beta }+\nu {a}^{\alpha \lambda }{a}^{\beta \mu }{\dot{a}}_{\lambda \mu },$$

where *ν* is the intra-membrane shear viscosity and the superscript dot $(\dot{\ast })$ refers to the time derivative. Since *a*_*αβ*_ = **a**_*α*_ · **a**_*β*_, we find

18 $${\dot{a}}_{\lambda \mu }=({{\bf{a}}}_{\lambda }\cdot {{\bf{a}}}_{\mu }\dot{)}={\dot{{\bf{a}}}}_{\lambda }\cdot {{\bf{a}}}_{\mu }+{{\bf{a}}}_{\lambda }\cdot {\dot{{\bf{a}}}}_{\mu }\mathrm{.}$$

In convected coordinates, ${\dot{{\bf{a}}}}_{\lambda }$ is defined as^17^ ${\dot{{\bf{a}}}}_{\lambda }=\partial {\bf{u}}{\boldsymbol{/}}\partial {\theta }^{\lambda }={{\bf{u}}}_{,\lambda }$ where ${\bf{u}}=\dot{{\bf{r}}}={v}^{\alpha }{{\bf{a}}}_{\alpha }+w{\bf{n}}$ is the velocity of a material point on the initial surface. It is now trivial to show that

19 $$\begin{array}{rcl}{\dot{{\bf{a}}}}_{\lambda } & = & {{\bf{u}}}_{,\lambda }={({v}^{\alpha }{{\bf{a}}}_{\alpha }+w{\bf{n}})}_{,\lambda }={v}_{,\lambda }^{\alpha }{{\bf{a}}}_{\alpha }+{v}^{\alpha }{{\bf{a}}}_{\alpha ,\lambda }+{w}_{,\lambda }{\bf{n}}+w{{\bf{n}}}_{,\lambda }\\  & = & ({v}_{\alpha ;\lambda }-w{b}_{\alpha \lambda }){{\bf{a}}}^{\alpha }+({v}^{\alpha }{b}_{\alpha \lambda }+{w}_{,\lambda }){\bf{n}},\,\because {v}_{\alpha ;\beta }={v}_{\alpha ,\beta }-{v}_{\beta }{{\rm{\Gamma }}}_{\alpha \beta }^{\lambda }\mathrm{.}\end{array}$$

Now, in view of Eqs (18 and 19), we find

20 $${\dot{a}}_{\lambda \mu }={v}_{\mu ;\lambda }+{v}_{\lambda ;\mu }-2w{b}_{\lambda \mu }$$

Further, from Eqs (10), (11), (17) and (20), the expression of the viscous stress can be derived as

21 $$\begin{array}{rcl}{\sigma }^{\beta \alpha } & = & (\lambda +W){a}^{\beta \alpha }-\mathrm{(2}{W}_{H}H+2{W}_{K}K){a}^{\beta \alpha }+{W}_{H}{\tilde{b}}^{\beta \alpha }\\  &  & +\,\nu [{a}^{\beta \lambda }{a}^{\alpha \mu }({v}_{\mu ;\lambda }+{v}_{\lambda ;\mu })-4wH{a}^{\beta \alpha }+2w{\tilde{b}}^{\beta \alpha }],\end{array}$$

where we also use the relations: *a*^*αλ*^*a*^*βμ*^*b*_*λμ*_ = *b*^*αβ*^ and ${b}^{\alpha \beta }=2H{a}^{\alpha \beta }-{\tilde{b}}^{\alpha \beta }$. The equation of motion (normal direction) of the lipid membrane in the presence of intra-membrane viscosity effects is therefore obtianed from Eqs (13), (15) and (21)

22 $$\begin{array}{rcl}p & = & -\,(\lambda +W){a}^{\beta \alpha }{b}_{\beta \alpha }+({W}_{H}H+{W}_{K}K){a}^{\beta \alpha }{b}_{\beta \alpha }-\frac{1}{2}{W}_{H}{\tilde{b}}^{\beta \alpha }{b}_{\beta \alpha }\\  &  & +\,{[\frac{1}{2}{({W}_{H})}_{;\beta }{a}^{\beta \alpha }+{({W}_{K})}_{;\beta }{\tilde{b}}^{\beta \alpha }]}_{;\alpha }-\nu [{a}^{\beta \lambda }{a}^{\alpha \mu }({v}_{\mu ;\lambda }+{v}_{\lambda ;\mu })\\  &  & -\,4wH{a}^{\beta \alpha }+2w{\tilde{b}}^{\beta \alpha }]{b}_{\beta \alpha }\mathrm{.}\end{array}$$

Utilizing the identities $H=\tfrac{1}{2}{a}^{\alpha \beta }{b}_{\alpha \beta }$, ${b}_{\beta \alpha }={b}_{\alpha }^{\mu }{a}_{\mu \beta }$, ${a}^{\alpha \lambda }{a}_{\lambda \beta }={\delta }_{\beta }^{\alpha }$ and ${a}^{\beta \alpha }K={b}_{\mu }^{\beta }{\tilde{b}}^{\mu \alpha }$ and knowing the fact that covariant derivatives of the dual metric and the covariant cofactor identically vanish $({\rm{i}}.\,{\rm{e}}{\rm{.}}\,{a}_{;\beta }^{\alpha \beta }=0,\,{\tilde{b}}_{;\beta }^{\alpha \beta }=0)$, the above equation further reduces to

23 $$\begin{array}{rcl}p & = & {W}_{H}(2{H}^{2}-K)+2H({W}_{K}K-W)-2\lambda H\\  &  & +\,{\rm{\Delta }}(\frac{1}{2}{W}_{H})+{({W}_{K})}_{;\alpha \beta }{\tilde{b}}^{\alpha \beta }-2\nu [\frac{1}{2}{b}^{\alpha \beta }({v}_{\alpha ;\beta }+{v}_{\beta ;\alpha })-2w(2{H}^{2}-K)],\end{array}$$

where Δ is the Laplace-Beltrami operator (i.e. Δ*ϕ* = *ϕ*_;*αβ*_*a*^*αβ*^), defined on the surface.

Similarly, by substituting Eqs (13) and (21) into Eq. (16), we obtain

24 $$\begin{array}{l}-\,({\gamma }_{,\alpha }+{W}_{K}{K}_{,\alpha }+{W}_{H}{H}_{,\alpha }){a}^{\beta \alpha }-4\nu w{H}_{,\alpha }{a}^{\beta \alpha }\\ \,+\,2\nu [\frac{1}{2}{a}^{\beta \lambda }{a}^{\alpha \mu }{({v}_{\mu ;\lambda }+{v}_{\lambda ;\mu })}_{;\alpha }-{w}_{,\alpha }{b}^{\beta \alpha }]=0,\end{array}$$

where ${({v}_{\mu ;\lambda }+{v}_{\lambda ;\mu })}_{;\alpha }={({v}_{\mu ;\lambda }+{v}_{\lambda ;\mu })}_{,\alpha }-({v}_{\beta ;\lambda }+{v}_{\lambda ;\beta }){{\rm{\Gamma }}}_{\mu \alpha }^{\beta }-({v}_{\beta ;\mu }+{v}_{\mu ;\beta }){{\rm{\Gamma }}}_{\lambda \alpha }^{\beta }$. Invoking ${b}^{\beta \alpha }={b}_{\lambda }^{\alpha }{a}^{\lambda \beta }$ and *γ*_,*α*_ = −*λ*_,*α*_ − *W*_*K*_*K*_,*α*_ − *W*_*H*_*H*_,*α*_, Eq. (24) further reduces to

$${a}^{\beta \alpha }[{\lambda }_{,\alpha }-4vw{H}_{,\alpha }+2\nu \{\frac{1}{2}{a}^{\lambda \mu }{({v}_{\mu ;\alpha }+{v}_{\alpha ;\mu })}_{;\lambda }-{w}_{,\lambda }{b}_{\alpha }^{\lambda }\}]=0.$$

Since *a*^*βα*^ ≠ 0, the above becomes

25 $${\lambda }_{,\alpha }-4vw{H}_{,\alpha }+2\nu [\frac{1}{2}{a}^{\lambda \mu }{({v}_{\mu ;\alpha }+{v}_{\alpha ;\mu })}_{;\lambda }-{w}_{,\lambda }{b}_{\alpha }^{\lambda }]=0,$$

which serves as the tangential equations of motion.

In the case of uniform membranes of the Helfrich type, the energy density *W* is defined by

26 $$W=k{H}^{2}+\bar{k}K,$$

where *k* and $\bar{k}$ are empirical constants (the bending moduli). It is noted here that, within the framework of the forgoing model, membranes with continuously distributed proteins can be accommodated through the energy density function: $W=k(\sigma ){H}^{2}+\bar{k}(\sigma )K;\sigma ({\theta }^{\alpha },t)$, where *σ*(*θ*^*α*^, *t*) describes the areal concentration of proteins on the membrane surface. However, the case is excluded from the present study in an effort to obtain mathematically tractable equations. Now, Eqs (23) and (26) yield

27 $$p=k[{\rm{\Delta }}H+2H({H}^{2}-K)]-2\lambda H-2[\nu \frac{1}{2}{b}^{\alpha \beta }({v}_{\alpha ;\beta }+{v}_{\beta ;\alpha })-2w(2{H}^{2}-K)],$$

while Eq. (25) remains intact. From the incompressibility condition $\dot{J}/J=\frac{1}{2}{a}^{\alpha \beta }{a}_{\alpha \beta }=0$^25^ together with Eqs (6) and (20), we also find

28 $${v}_{;\alpha }^{\alpha }-2wH=0.$$

Finally, admissible boundary conditions (e.g., boundary forces **f** and moments *M* on ∂*ω*) of Eq. (27) are derived in detail in^9^ and^15^. In the case of uniform viscous Helfrich membranes, these are given by

29 $$\begin{array}{rcl}M & = & \frac{1}{2}k({\kappa }_{\nu }+{\kappa }_{\tau })+\bar{k}\tau ,\,{f}_{n}=\bar{k}\tau ^{\prime} -k{H}_{,\nu }\\ {f}_{\nu } & = & \frac{1}{4}k({\kappa }_{\nu }^{2}-{\kappa }_{\tau }^{2})-\bar{k}{\tau }^{2}+\lambda \\  &  & +\,\nu [{a}^{\beta \lambda }{a}^{\alpha \mu }({v}_{\mu ;\lambda }+{v}_{\lambda ;\mu })-4wH{a}^{\beta \alpha }+2w{\tilde{b}}^{\beta \alpha }]{\nu }_{\beta }{\nu }_{\alpha },\\ {f}_{\tau } & = & \nu [{a}^{\beta \lambda }{a}^{\alpha \mu }({v}_{\mu ;\lambda }+{v}_{\lambda ;\mu })-4wH{a}^{\beta \alpha }\\  &  & +\,2w{\tilde{b}}^{\beta \alpha }]{\nu }_{\beta }{\nu }_{\alpha }-\frac{1}{2}k\tau {\kappa }_{\nu }-(\frac{1}{2}k+\bar{k})\tau {\kappa }_{\tau },\end{array}$$

and corner forces

30 $${{\bf{f}}}_{i}=\bar{k}{[\tau ]}_{i}{\bf{n}},$$

where

31 $${\kappa }_{\nu }={b}_{\alpha \beta }{\nu }^{\alpha }{\nu }^{\beta },\,{\kappa }_{\tau }={b}_{\alpha \beta }{\nu }^{\alpha }{\nu }^{\beta },\,\tau ={b}_{\alpha \beta }{\nu }^{\alpha }{\tau }^{\beta }$$

are the normal curvatures of *ω* in the direction of *ν* and *τ* and *τ* is the twist of *w* on the (*ν*, *τ*) axes with *τ* = **n** × *ν*. We note here that the normal force remains intact in the presence of intra-membrane viscosity effects.

## Monge Representation and Superposed Incremental Deformations

In order to study the responses of the membrane, we use the Monge representation with space vector **r**(*θ*^*α*^, *t*) representing material points on the membrane surface *w*, which is given by

32 $${\bf{r}}({{\boldsymbol{\theta }}}^{\alpha },t)={\boldsymbol{\theta }}+z({\boldsymbol{\theta }},t){\bf{k}},$$

where ***θ***(*θ*^*α*^) is position on a plane defined by the unit normal **k** and *z*(***θ***, *t*) is height function that describes the bilayer membrane mid-plane shape. The Monge representation is an approximation of out-of-plane deformations in which no folds of the membrane are allowed, and hence, *z*(***θ***, *t*) is restricted to a single-valued function. For instance, the membrane surface can be represented by orthonormal Cartesian basis ***θ*** = *θ*^*α*^**e**_*α*_ and, unless otherwise specified, the subscripts of the surface coordinates are dropped and replaced by 1 = *x*, 2 = *y* for convenience. Within this setting, we compute

33 $$\begin{array}{rcl}{{\bf{r}}}_{,t} & = & {z}_{,t}{\bf{k}},\,{{\bf{a}}}_{\alpha }={{\bf{e}}}_{\alpha }+{z}_{,\alpha }{\bf{k}},\\  &  & \,\,\,\,\,\,a={\rm{\det }}({a}_{\alpha \beta })\\  &  & \,\,\,\,\,\,\,\,=[1+{({z}_{,1})}^{2}+{({z}_{,2})}^{2}],\\  &  & \,\,{a}^{\alpha \beta }={\delta }_{\alpha \beta }+{z}_{,\alpha }{z}_{,\beta },\\ H & = & \frac{(1+{z}_{\mathrm{,2}}^{2}){z}_{\mathrm{,11}}+(1+{z}_{\mathrm{,1}}^{2}){z}_{\mathrm{,22}}-2{z}_{\mathrm{,1}}{z}_{\mathrm{,2}}{z}_{\mathrm{,12}}}{2{a}^{\mathrm{3/2}}},\,K=\frac{({z}_{\mathrm{,11}}{z}_{\mathrm{,22}}-{z}_{\mathrm{,12}}^{2})}{{a}^{2}},\\ {\bf{n}} & = & \frac{({\bf{k}}-\nabla z)}{\sqrt{a}}\,{\rm{and}}\,{\bf{b}}=\frac{{z}_{,\alpha \beta }}{\sqrt{a}}({{\bf{a}}}^{\alpha }\otimes {{\bf{a}}}^{\beta }),\end{array}$$

where is *δ*_*αβ*_ Kronecker delta, $\nabla z={z}_{,\alpha }{{\bf{e}}}_{\alpha }$ is the gradient evaluated on the surface and is **b** curvature tensor with components ${b}_{\alpha \beta }={z}_{,\alpha \beta }/\sqrt{a}$,

34 $$\begin{array}{rcl}{{\bf{a}}}^{1} & = & \frac{1}{a}[(1+{z}_{\mathrm{,2}}^{2})\,({{\bf{e}}}_{1}+{z}_{\mathrm{,1}}{\bf{k}})-{z}_{\mathrm{,1}}{z}_{\mathrm{,2}}({{\bf{e}}}_{2}+{z}_{\mathrm{,2}}{\bf{k}})],\,{\rm{and}}\\ {{\bf{a}}}^{2} & = & \frac{1}{a}[(1+{z}_{\mathrm{,1}}^{2})\,({{\bf{e}}}_{2}+{z}_{\mathrm{,2}}{\bf{k}})-{z}_{\mathrm{,1}}{z}_{\mathrm{,2}}({{\bf{e}}}_{1}+{z}_{\mathrm{,1}}{\bf{k}})]\mathrm{.}\end{array}$$

Further, the normal velocity and the Christoffel symbols are computed as

35 $$w={{\bf{r}}}_{,t}\cdot {\bf{n}}={z}_{,t}/\sqrt{a},\because {{\bf{r}}}_{,t}=w{\bf{n}},$$

and

36 $${{\rm{\Gamma }}}_{\alpha \beta }^{\lambda }={z}_{,\lambda }{z}_{,\alpha \beta }/\sqrt{a},$$

respectively.

The evaluation of the resulting shape equation Eq. (27) in terms of Eqs (33–36) furnishes a highly nonlinear PDE system, which most often requires heavy computational resources. Instead, a means of ‘admissible linearization’ can be employed to make the system mathematically tractable with minimum loss of generality. The concept of the superposed incremental deformations has been widely and successfully adopted in the relevant subject of studies (see, for example^15,26–28^). Within this prescription, the derivatives of *z*(*θ*, *t*) of all orders are considered to be ‘small’ (e.g. ${z}_{,\alpha }\ll 1.$), and therefore, their products can be neglected. Accordingly, using the notation ‘$\simeq$’ to identify equations to the leading order in *z*, we have

37 $$\begin{array}{l}a\simeq \mathrm{1,}\,w\simeq {z}_{t},\,{\bf{n}}={\bf{k}}-{\nabla }_{p}z,\,{{\bf{a}}}^{\alpha }\simeq {{\bf{a}}}_{\alpha }={{\bf{e}}}_{\alpha }+{z}_{,\alpha }{\bf{k}},\,{{\rm{\Gamma }}}_{\alpha \beta }^{\lambda }\simeq \mathrm{0,}\,{\bf{n}}\cdot {\bf{r}}\simeq z-{z}_{,\alpha }{\theta }^{\alpha },\\ {\bf{b}}\simeq {\nabla }_{p}^{2}z,\,H\simeq \frac{1}{2}{{\rm{\Delta }}}_{p}z\,{\rm{and}}\,K\simeq \mathrm{0,}\end{array}$$

where the subscript ${(\ast )}_{p}$ refers to the projected counterparts of $(\,\ast \,)$ on the coordinate plane *ω*_*p*_, ${\nabla }_{p}^{2}z={z}_{,\alpha \beta }$ ${{\bf{e}}}_{\alpha }\otimes {{\bf{e}}}_{\beta }$ is the second gradient and ${{\rm{\Delta }}}_{p}z=tr({\nabla }_{p}^{2}z)$ is the corresponding Laplacian, respectively. In particular, the straining (20), viscous stress (17) and incompressibility condition (28) can be approximated as:

38 $${\dot{a}}_{\alpha \beta }\simeq {v}_{\alpha ,\beta }+{v}_{\beta ,\alpha },$$

39 $${\sigma }^{\alpha \beta }\simeq -\,\frac{1}{2}\gamma {\delta }_{\alpha \beta }+\nu ({v}_{\alpha ,\beta }+{v}_{\beta ,\alpha }),$$

and

40 $${v}_{\alpha ,\alpha }\simeq 0.$$

Further, the equations of motion in normal and tangential directions (Eqs (25) and (27)) can be approximated as

41 $$\frac{1}{2}k{{\rm{\Delta }}}_{p}({{\rm{\Delta }}}_{p}z)-\lambda {{\rm{\Delta }}}_{p}z-\nu {z}_{,\alpha \beta }({v}_{\alpha ,\beta }+{v}_{\beta ,\alpha })\simeq p,\,{\rm{and}}\,{\lambda }_{,\alpha }+v{({v}_{\lambda ,\alpha }+{v}_{\alpha ,\lambda })}_{,\lambda }\simeq 0.$$

In view of, Eqs (38) and (40) (e.g. *z*_,*αβ*_ = *z*_,*βα*_, ${v}_{\lambda ,\alpha \lambda }={v}_{\lambda ,\lambda \alpha }\ldots$), the above are equivalent to

42 $$\frac{1}{2}k{{\rm{\Delta }}}_{p}({{\rm{\Delta }}}_{p}z)-\lambda {{\rm{\Delta }}}_{p}z-2\nu {z}_{,\alpha \beta }{v}_{\alpha ,\beta }\simeq p\,{\rm{and}}\,{\lambda }_{,\alpha }+v{{\rm{\Delta }}}_{p}{v}_{,\alpha }\simeq 0.$$

To obtain simplified edge conditions, let **r**(*S*, *t*) = **r**(***θ***(*S*), *t*), where ***θ***(*S*) is the arc length parameterization of the projected curve ∂*ω*_*p*_ on the plane *ω*_*p*_. Thus, under the Monge representation, we obtain

43 $${\bf{r}}(S)^{\prime} =\frac{d{\bf{r}}}{dS}=\frac{d({\boldsymbol{\theta }}+z({\boldsymbol{\theta }}){\bf{k}})}{dS}=\frac{d{\boldsymbol{\theta }}}{dS}+(\frac{z({\boldsymbol{\theta }})}{d{\boldsymbol{\theta }}}\cdot \frac{d{\boldsymbol{\theta }}}{dS}){\bf{k}}={\tau }_{p}+({\nabla }_{p}z\cdot {\tau }_{p}){\bf{k}};\frac{d{\boldsymbol{\theta }}}{dS}\equiv {\tau }_{p}\mathrm{.}$$

Eq. (43) is equivalent to

44 $${\bf{r}}(S)^{\prime} =\frac{d{\bf{r}}(s)}{ds}\frac{ds}{dS}=\tau |{\bf{r}}(s)^{\prime} |;\frac{d{\bf{r}}(s)}{ds}\equiv \tau \,{\rm{and}}\,\frac{ds}{dS}\equiv |{\bf{r}}(s)^{\prime} \mathrm{|.}$$

Accordingly, Eqs (43 and 44). yield

45 $${\bf{r}}(S)^{\prime} =\tau |{\bf{r}}(s)^{\prime} |={\tau }_{p}+({\nabla }_{p}z\cdot {\tau }_{p}){\bf{k}},\,{\rm{and}}$$

46 $$|{\bf{r}}(s)^{\prime} |=|{\tau }_{p}+({\nabla }_{p}z\cdot {\tau }_{p}){\bf{k}}|=\sqrt{1+{({\nabla }_{p}z\cdot {\tau }_{p})}^{2}}\cong 1.$$

Comparing the right sides of Eqs (45 and 46) and referring Eq. (37), we have

47 $$\tau \cong {\tau }_{p}+({\nabla }_{p}z\cdot {\tau }_{p}){\bf{k}},\,{\rm{and}}$$

48 $$\nu =\tau \times {\bf{n}}\simeq [{\tau }_{p}+({\nabla }_{p}z\cdot {\tau }_{p}){\bf{k}}]\times ({\bf{k}}-{\nabla }_{{\bf{p}}}{\bf{z}})={\nu }_{p}+{\nabla }_{p}z\times {\tau }_{p},$$

where *ν*_*p*_ = *τ*_*p*_ × **k** is the unit normal to the projected curve. Consequently,

49 $${\nu }_{\alpha }={{\bf{a}}}_{\alpha }\cdot \nu \simeq {({\nu }_{\alpha })}_{p}+{e}_{\alpha \beta }{z}_{,\beta }\,{\rm{and}}\,{\tau }_{\alpha }={{\bf{a}}}_{\alpha }\cdot \tau \simeq {({\tau }_{\alpha })}_{p},$$

where (*ν*_*α*_)_*p*_ = **e**_*α*_ · **v**_*p*_ and (*ν*_*α*_)_*p*_ = **e**_*α*_ · *τ*_*p*_ and *e*_*αβ*_ is the unit alternator defined by *e*_12_ = −*e*_21_ = 1 and *e*_11_ = *e*_22_ = 0. Also, invoking Eqs (31), (37), (47) and (48), becomes

50 $${\kappa }_{\nu }=\nu \cdot {\bf{b}}\nu \simeq ({\nu }_{p}+{\nabla }_{p}z\times {\tau }_{p})\cdot {\nabla }_{p}^{2}z({\nu }_{p}+{\nabla }_{p}z\times {\tau }_{p})\simeq {\nu }_{p}\cdot ({\nabla }_{p}^{2}z){\nu }_{p},$$

and similarly for

51 $${\kappa }_{\tau }\simeq {\tau }_{p}\cdot ({\nabla }_{p}^{2}z){\tau }_{p}\,{\rm{and}}\,\tau \simeq {\tau }_{p}\cdot ({\nabla }_{p}^{2}z){\nu }_{p}.$$

Therefore, it follows from Eqs (29)_1–3_ and (49–51) that

52 $$M\simeq \frac{1}{2}k{{\rm{\Delta }}}_{p}z+\bar{k}{\tau }_{p}\cdot ({\nabla }_{p}^{2}z){\tau }_{p},\,{f}_{\nu }\simeq \lambda +\nu ({v}_{\alpha ,\beta }+{v}_{\beta ,\alpha }){\nu }_{\beta }{\nu }_{\alpha }\,{\rm{and}}\,{f}_{\tau }\simeq \nu ({v}_{\alpha ,\beta }+{v}_{\beta ,\alpha }){\tau }_{\beta }{\nu }_{\alpha },$$

and

53 $${\nu }_{\alpha }{\nu }_{\beta }\simeq {({\nu }_{\alpha }{\nu }_{\beta })}_{p}+{e}_{\alpha \lambda }{z}_{,\lambda }{({\nu }_{\beta })}_{p}+{e}_{\beta \lambda }{z}_{,\lambda }{({\nu }_{\alpha })}_{p}\,{\rm{and}}\,{\tau }_{\beta }{\nu }_{\alpha }\simeq {({\tau }_{\beta }{\nu }_{\alpha })}_{p}+{e}_{\alpha \lambda }{z}_{,\lambda }{\tau }_{\beta }.$$

Now, the normal force is given by

54 $${f}_{n}=\overline{k}\tau ^{\prime} (s)-k{H}_{,\nu },$$

where ${(\ast )}_{,\nu }$ is the normal derivatives on ∂*ω*. By Eq. (46), the arclength derivatives satisfies the approximation

55 $$\tau ^{\prime} (S)=\frac{d\tau (s)}{ds}\frac{ds}{dS}=\tau ^{\prime} (s)|{\bf{r}}(s)^{\prime} |\simeq \tau ^{\prime} (s\mathrm{).}$$

Here $\tau ^{\prime} (S)=\frac{d\tau }{dS}=\frac{d\tau }{d{\boldsymbol{\theta }}}\cdot \frac{d{\boldsymbol{\theta }}}{dS}={\nabla }_{p}\tau \cdot {\tau }_{p}$ is the arclength derivative on the projected curve. In addition, *H*_,*ν*_ can be re-written as

56 $${H}_{,\nu }=(\frac{\partial H}{\partial {\theta }^{\alpha }})\,(\frac{\partial {\theta }^{\alpha }}{\partial {\bf{r}}})\cdot \frac{\partial {\bf{r}}}{\partial \nu }={{\bf{a}}}^{\alpha }{H}_{,\alpha }\cdot (\frac{\partial {\bf{r}}}{\partial {\theta }^{\alpha }})\,(\frac{\partial {\theta }^{\alpha }}{\partial \nu })={{\bf{a}}}^{\alpha }{H}_{,\alpha }\cdot {{\bf{a}}}_{\beta }{v}^{\beta }=\nu \cdot {{\bf{a}}}^{\alpha }{H}_{,\alpha },$$

Consequently, by substituting Eqs (37) and (48) into Eq. (56), the leading order expression of *H*_,*α*_ can be evaluated on the projected plane as

57 $$\begin{array}{rcl}{H}_{,\nu } & = & \nu \cdot {{\bf{a}}}^{\alpha }{H}_{,\alpha }=[{\nu }_{p}+{\nabla }_{p}z\times {\tau }_{p}+\ldots ]\cdot [{{\bf{e}}}_{\alpha }+{z}_{,\alpha }{\bf{k}}+\ldots ]{H}_{,\alpha }\\  & \simeq  & {\nu }_{p}\cdot ({{\bf{e}}}_{\alpha }{H}_{,\alpha })={\nu }_{p}\cdot {\nabla }_{p}H,\end{array}$$

and therefore, we obtain

58 $${f}_{n}\simeq \overline{k}{\nabla }_{p}\tau \cdot {{\boldsymbol{\tau }}}_{p}-k{{\boldsymbol{\nu }}}_{p}\cdot {\nabla }_{p}H.$$

## Solutions to the Linearized Systems

Consider the case where the membrane flows over a rectangular portion of the plane $(\frac{-\,a}{2}\le x\le \frac{a}{2},\frac{-\,b}{2}\le y\le \frac{b}{2})$. Driven by the controlled force, lipid molecules are flowing in through the boundary, while the boundary remains clamped. The corresponding kinematic edge conditions are

59 $$z=0\,{\rm{and}}\,{\bf{n}}={\bf{k}}.$$

In view of Eq. (37), the later implies that $\nabla z=0$ and thus both *z*_,*α*_ and the normal derivative *z*_,*ν*_ = *ν*^*α*^*z*_,*α*_ vanish on the boundary. Accordingly, the edge moment acting on the boundary Eq. (52)_1_ becomes

60 $$M\simeq \frac{1}{2}k{{\rm{\Delta }}}_{p}z\mathrm{.}$$

It is suggested that the kinetic conditions lead to the particular set of the tangential and normal force (i.e. *f*_*τ*_ = 0, *f*_*ν*_ = −*q*), where *q* is prescribed surface pressure (see, for example^9^ and^29^). Thus, Eq. (52)_2–3_ furnishes

61 $$({v}_{\alpha ,\beta }+{v}_{\beta ,\alpha })\,{({\tau }_{\beta }{\nu }_{\alpha })}_{p}\simeq 0\,{\rm{and}}\,-\,(q+\lambda )\simeq \nu ({v}_{\alpha ,\beta }+{v}_{\beta ,\alpha })\,{({\nu }_{\beta }{\nu }_{\alpha })}_{p},$$

where the approximations have been made via Eq. (53). We note here that, up to leading order, membrane shape has negligible effects on the surface flow, whereas shape is influenced by the flow via the viscosity term in the shape equation (41). Admissible linearization thus reduces the non-linear, fully coupled equations to a system of PDEs with one-way coupling. Further, the present model incorporates the purely elastic theory of lipid membranes in the limit of vanishing viscosity $\nu \simeq 0$;

62 $$\frac{1}{2}k{{\rm{\Delta }}}_{p}({{\rm{\Delta }}}_{p}z)-\lambda {{\rm{\Delta }}}_{p}z\simeq p,$$

while the kinetic boundary conditions remain intact (i.e. *z* = 0 and ${\nabla }_{p}z=0$). This observation, in turn, suggests that the decay of a dynamic solution to a purely elastic solution can be investigated by assigning *q*(*t*) on the boundary in a way that

63 $$q({\theta }^{\alpha },t)=q(t)\,{\rm{and}}\,\bar{\lambda }=\lambda +q,$$

where *q*(*θ*^*α*^, *t*) is understood as a uniform function assigned in the interior (i.e. *q*(*θ*^*α*^, *t*) = *q*(*θ*^*α*^)) at each sequential step. Then the equations of motion Eq. (42) and boundary conditions Eq. (61) become

64 $$\frac{1}{2}k{{\rm{\Delta }}}_{p}({{\rm{\Delta }}}_{p}z)-\lambda {{\rm{\Delta }}}_{p}z-2\nu {z}_{,\alpha \beta }{v}_{\alpha ,\beta }\simeq p,\,{\bar{\lambda }}_{,\alpha }+v{{\rm{\Delta }}}_{p}{v}_{,\alpha }\simeq 0,\,{v}_{\alpha ,\alpha }\simeq 0,$$

and

65 $$({v}_{\alpha ,\beta }+{v}_{\beta ,\alpha })\,{({\tau }_{\beta }{\nu }_{\alpha })}_{p}\simeq 0\,{\rm{and}}\,-\,\bar{\lambda }\simeq \nu ({v}_{\alpha ,\beta }+{v}_{\beta ,\alpha })\,{({\nu }_{\beta }{\nu }_{\alpha })}_{p},$$

respectively, where *q* is prescribed.

Within the domain of interest (a rectangular portion), we now create a particular set of intra-surface viscous flow as

66 $${v}_{x}=-\,Ax\,{\rm{and}}\,{v}_{y}=Ay.$$

Since *v*_*x*,*x*_ + *v*_*y*,*y*_ = *A* − *A* = 0 and either *τ*_*x*_*ν*_*y*_ or *τ*_*y*_*ν*_*x*_ vanishes on the boundary (e.g. for $x=\frac{a}{2}$ boundary, *ν*_*p*_ = −*v*_*x*_**e**_1_ and *τ*_*p*_ = −*τ*_*y*_**e**_2_ so that *v*_*y*_ = *τ*_*x*_ = 0), Eqs (64)_3_ and (65)_1_ are automatically satisfied. Similarly, it follows from Eqs (65 and 66) that

67 $$\bar{\lambda }\simeq 0\,{\rm{and}}\,\lambda \simeq -\,q,$$

which agree with the elastic boundary condition in the limit as discussed in^25–29^ (i.e. *λ* = −*q*). In addition, Eq. (64)_2_ is also satisfied because both ${\bar{\lambda }}_{,\gamma }$ and Δ*v*_*γ*_ vanish identically (e.g. Δ*v*_*x*_ = Δ(*Ax*) = 0 and ${\bar{\lambda }}_{,x}=0.$). Consequently, the systems of coupled PDEs (64-65) now reduces to

68 $$\frac{1}{2}k{{\rm{\Delta }}}_{p}({{\rm{\Delta }}}_{p}z)-\lambda {{\rm{\Delta }}}_{p}z-2\nu {z}_{,\alpha \beta }{v}_{\alpha ,\beta }\simeq p,z=0,\nabla z=0\,{\rm{and}}\,\frac{1}{2}k{{\rm{\Delta }}}_{p}({{\rm{\Delta }}}_{p}z)=M\,{\rm{on}}\,\partial {\omega }_{p}.$$

In the case of vanishing *p*, the solution of the above is obtained by^30^

69 $$\begin{array}{rcl}z(x\mathrm{,\ }y) & = & \sum _{n\mathrm{=1}}^{\infty }\,\{({A}_{n}\,\cosh \,\sqrt{-\,P+\sqrt{{P}^{2}-4Q}}x\\  &  & +\,{B}_{n}\,\cosh \,\sqrt{-\,P-\sqrt{{P}^{2}-4Q}}x)\,\cos \,\beta y\\  &  & +\,({C}_{n}\,\cosh \,\sqrt{-\,T+\sqrt{{T}^{2}-4U}}y\\  &  & +\,{D}_{n}\,\cosh \,\sqrt{-\,T-\sqrt{{T}^{2}-4U}}y)\,\cos \,\alpha x\},\end{array}$$

where

70 $$\begin{array}{rcl}\alpha  & = & \frac{n\pi }{a},\,\beta =\frac{n\pi }{b},\,P=-\,{\beta }^{2}+{\mu }^{2}+\frac{4A\nu }{k},\,Q={\beta }^{4}-({\mu }^{2}+\frac{4A\nu }{k}){\beta }^{2},\\ T & = & -\,{\alpha }^{2}+{\mu }^{2}+\frac{4A\nu }{k}\,{\rm{and}}\,U={\alpha }^{4}-({\mu }^{2}-\frac{4A\nu }{k}){\alpha }^{2}\mathrm{.}\end{array}$$

**Figure 1:**
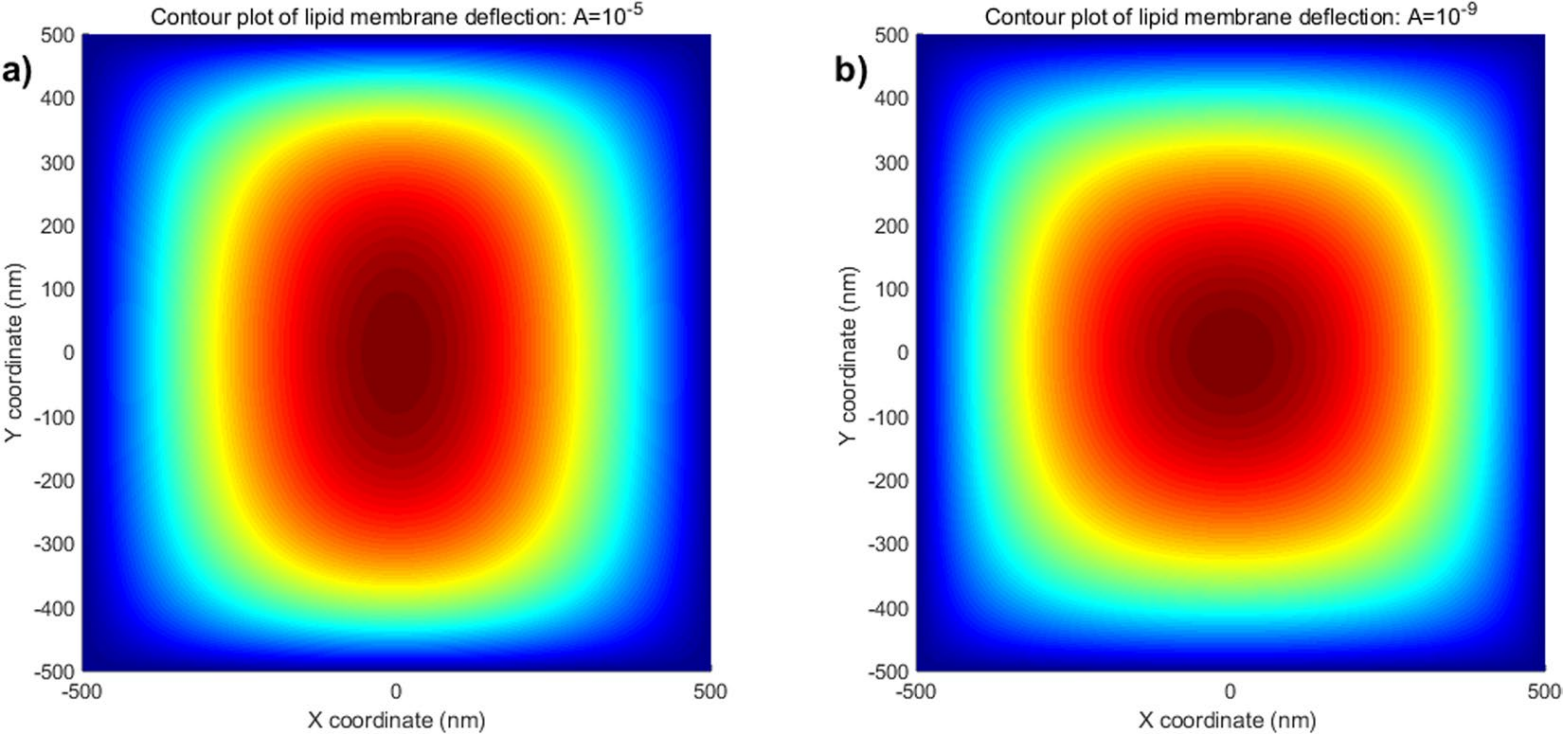
Membrane shape evolutions in the presence of intra-membrane viscosity: *A* = 10^−5^ and 10^−9^.

**Figure 2:**
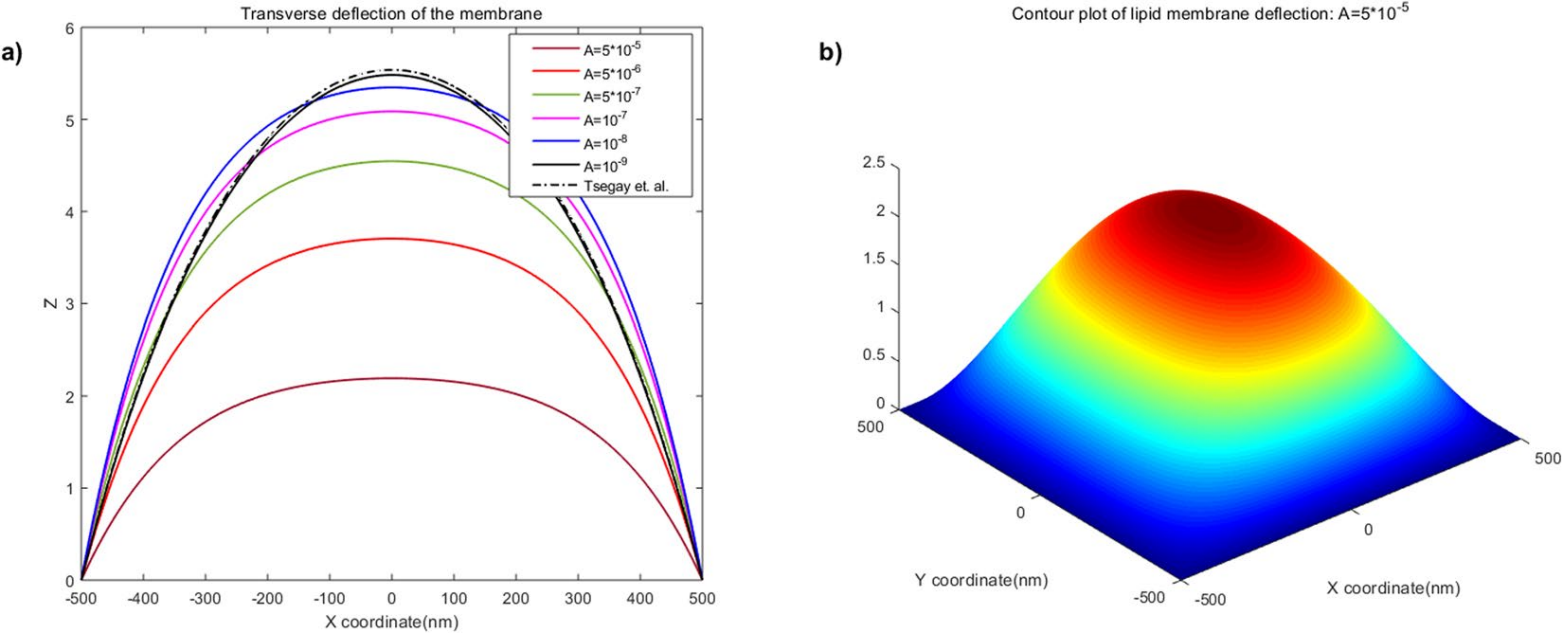
Deflections of lipid membrane with respect to intra-membrane viscous flows.

By imposing boundary conditions (68)_2–4_, the unknown constants (e.g. *A*_*n*_, *B*_*n*_ etc…) can be completely determined. Here, we omit details for the sake of conciseness which can be found in^16^. The value of intramembrane surface viscosity *ν* = 10^−4^ *pN* · *s*/*nm* and the bending modulus of the membrane *k* = 82 *pN* · *nm* are adopted from the work of^31^ and^32^, respectively. We also note that the data are obtained under the normalized setting unless otherwise specified. It is clear from Fig. 1 that membrane shape is influenced by the viscous flow via the viscosity term in the shape equation (64)_1_. More precisely, the applied flow gives rise to straining effects on the membrane shape (Fig. 1) in lateral direction. The corresponding transverse deflections (Fig. 2) decrease with the increasing velocity field of viscous flow. In addition, Fig. 2a illustrates that the obtained solutions accommodate those presented in^16^ in the limit of vanishing viscous flow. This can also be seen by the reduction of Eq. (64)_1_ to the classical shape equation (see, for example^15,16^) in the absence of viscous flow (i.e. *v*_*α*_ = 0) and so too the corresponding solutions.

## Membrane-Substrate Interactions in the Presence of Viscous Flow

In light of the foregoing discussions, we now investigate a membrane-substrate interaction problem under the influence of intra-membrane viscosity. Driven by the bi-normal (transverse direction) force *f*_*n*_, the interaction occurs on the boundary *r* = *a* of an annular portion of the membrane, while it remains clamped (i.e. *z* = 0 and *n* = **k**). Under a polar-coordinate parametrization of the Monge plane, Eq. (32) is replaced by

71 $${\bf{r}}({\theta }^{\alpha },t)=r{{\bf{e}}}_{r}(\theta )+z(r,\theta ,t){\bf{k}},$$

where *r* is the radius and *θ* is the azimuth; **e**_*r*_ is the usual radial unit vector at azimuth *θ*. We take {*θ*^1^, *θ*^2^} = {*r*, *θ*} and recast Eq. (33) as

72 $$\begin{array}{rcl}{{\bf{r}}}_{,t} & = & {z}_{,t}{\bf{k}},\,{{\bf{a}}}_{1}={{\bf{e}}}_{r}(\theta )+{z}_{,r}(r,\theta ){\bf{k}},\\  &  & \,\,\,{{\bf{a}}}_{2}=r{{\bf{e}}}_{\theta }(\theta )+{z}_{,\theta }(r,\theta ){\bf{k}},\\  &  & \,\,\,\,\,a={\rm{\det }}({a}_{\alpha \beta })\\  &  & \,\,\,\,\,\,\,\,={r}^{2}\mathrm{(1}+{z}_{,r}^{2})+{z}_{,\theta }^{2},\\ {a}^{11} & = & \frac{{r}^{2}+{z}_{,\theta }^{2}}{a},\,{a}^{22}=\frac{1+{z}_{,r}^{2}}{a},\,{a}^{12}={a}^{21}=-\,\frac{{z}_{,r}{z}_{,\theta }}{a},\\ H & = & \frac{1}{2{a}^{\mathrm{3/2}}}[{r}^{3}{z}_{,rr}+{r}^{2}{z}_{,r}+{r}^{2}{z}_{,r}^{3}+r{z}_{,rr}{z}_{,\theta }^{2}-2r{z}_{,r\theta }{z}_{,r}{z}_{,\theta }+r{z}_{,\theta \theta }+r{z}_{,\theta \theta }{z}_{,r}^{2}+2{z}_{,r}{z}_{,\theta }^{2}],\\ K & = & \frac{1}{{a}^{2}}[{r}^{3}{z}_{,r}{z}_{,rr}+{r}^{2}{z}_{,rr}{z}_{,\theta \theta }-{r}^{2}{z}_{,r\theta }^{2}+2r{z}_{,r\theta }-{z}_{,\theta }^{2}]\,{\rm{and}}\\  &  & {\bf{n}}=\frac{1}{\sqrt{a}}(\,-\,{z}_{,\theta }{{\bf{e}}}_{\theta }-r{z}_{,r}{{\bf{e}}}_{r}+r{\bf{k}}\mathrm{).}\end{array}$$

Further, the normal velocity and the curvature tensor are given by

73 $$w={{\bf{r}}}_{,t}\cdot {\bf{n}}=r{z}_{,t}/\sqrt{a}\,{\rm{and}}\,{\bf{b}}={b}_{\alpha \beta }({{\bf{a}}}^{\alpha }\otimes {{\bf{a}}}^{\beta }),$$

where

74 $$\begin{array}{rcl}{b}_{11} & = & {z}_{,rr}{\bf{k}},\,{b}_{12}={b}_{21}=\frac{1}{\sqrt{a}}(r{z}_{,r\theta }-{z}_{,\theta }),\,{b}_{22}=\frac{1}{\sqrt{a}}({r}^{2}{z}_{,r}+r{z}_{,\theta \theta }),\\ {{\bf{a}}}^{1} & = & \frac{1}{a}[({r}^{2}+{z}_{,\theta }^{2}){{\bf{e}}}_{r}-r{z}_{,r}{z}_{,\theta }{{\bf{e}}}_{\theta }+{r}^{2}{z}_{,r}{\bf{k}}],\,{\rm{and}}\\ {{\bf{a}}}^{2} & = & \frac{1}{a}[\,-\,r{z}_{,r}{z}_{,\theta }{{\bf{e}}}_{r}+r\mathrm{(1}+{z}_{,r}^{2}){{\bf{e}}}_{\theta }+{z}_{,\theta }{\bf{k}}\mathrm{].}\end{array}$$

By extracting leading order terms, Eqs (72–74) yield

75 $$\begin{array}{rcl}a & \simeq  & {r}^{2},\,w\simeq r{z}_{,t},\,{\bf{n}}\simeq {\bf{k}}-{\nabla }_{p}z,\,{{\bf{a}}}^{1}\simeq {{\bf{e}}}_{r}+{z}_{,r}{\bf{k}},\,{{\bf{a}}}^{2}\simeq \frac{1}{r}{{\bf{e}}}_{\theta }+\frac{1}{{r}^{2}}{z}_{,\theta }{\bf{k}}\\ {\bf{b}} & \simeq  & {\nabla }_{p}^{2}z,\,H\simeq \frac{1}{2}{{\rm{\Delta }}}_{p}z\,{\rm{and}}\,K\simeq \mathrm{0,}\end{array}$$

and the incompressibility condition Eq. (28) becomes

76 $$0\simeq {v}_{\alpha ,\alpha }={\rm{div}}\,v={v}_{r,r}+\frac{{v}_{\theta ,\theta }}{r}+\frac{{v}_{r}}{r}.$$

The equations of motion (42) and boundary conditions (61) are approximated as

77 $$\frac{1}{2}k{{\rm{\Delta }}}_{p}({{\rm{\Delta }}}_{p}z)-\lambda {{\rm{\Delta }}}_{p}z-2\nu {z}_{,\alpha \beta }{v}_{\alpha ,\beta }\simeq p,\,{\bar{\lambda }}_{,\alpha }+v{{\rm{\Delta }}}_{p}{v}_{,\alpha }\simeq \mathrm{0,}\,{v}_{\alpha ,\alpha }\simeq \mathrm{0,}$$

and

78 $$({v}_{\alpha ,\beta }+{v}_{\beta ,\alpha })\,{({\tau }_{\beta }{\nu }_{\alpha })}_{p}\simeq 0\,{\rm{and}}\,-\,\bar{\lambda }\simeq \nu ({v}_{\alpha ,\beta }+{v}_{\beta ,\alpha })\,{({\nu }_{\beta }{\nu }_{\alpha })}_{p}\mathrm{.}$$

Lastly, on the interacting boundary, we have from^15^ and^28^ that

79 $$\sigma ={f}_{n}\simeq \overline{k}{\nabla }_{p}\tau \cdot {{\boldsymbol{\tau }}}_{p}-k{{\boldsymbol{\nu }}}_{p}\cdot {\nabla }_{p}H,$$

where *σ* is an empirical wetting constant. In the present case (i.e. *ν*_*p*_ = −**e**_*r*_ and *τ*_*p*_ = **e**_*θ*_), the above condition reduces to

80 $${({{\rm{\Delta }}}_{p}z)}_{,r}=\frac{2\sigma }{k}.$$

### Example 1: Circumferentially dominant viscous flow

**Figure 3:**
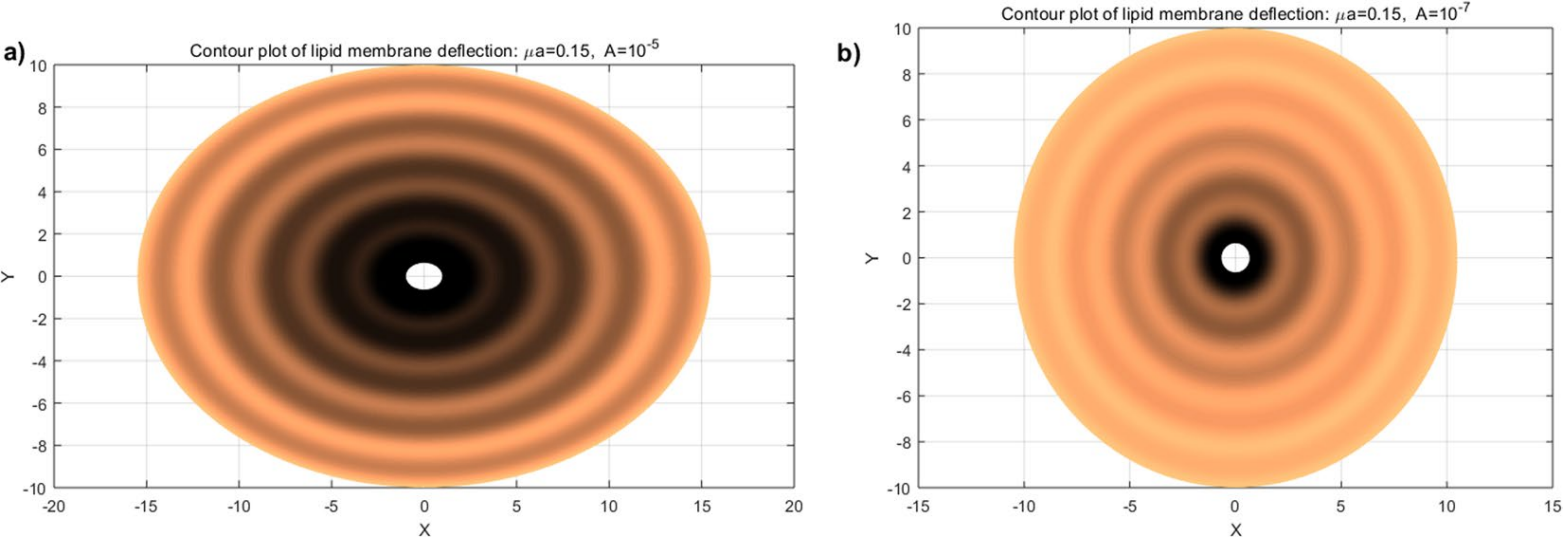
Membrane shape evolutions with intra-membrane viscosity (Circumferential): *A* = 10^−5^ and 10^−7^.

**Figure 4:**
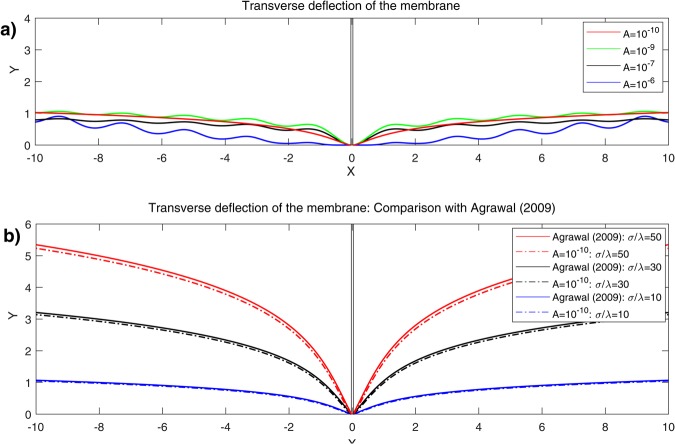
Deflections of lipid membrane with respect to intra-membrane viscous flows.

We consider a case in which

81 $${v}_{\theta }=A\,{\rm{and}}\,{v}_{r}=\frac{A}{r},$$

so that (77)_2–3_ are met and thus yield $\bar{\lambda }\simeq 0$ and $\lambda \simeq -\,q$. Accordingly, in the case of vanishing *p*, Eqs (77 and 78) become

82 $$\begin{array}{l}({z}_{,rrrr}+\frac{2{z}_{,rrr}}{r}+\frac{{z}_{,rr}}{{r}^{2}}+\frac{2{z}_{,rr\theta \theta }}{{r}^{2}}+\frac{2{z}_{,r\theta \theta }}{{r}^{3}}+\frac{{z}_{,\theta \theta \theta \theta }}{{r}^{4}})\\ \,-\,{\mu }^{2}({z}_{,rr}+\frac{{z}_{,r}}{r}+\frac{{z}_{,\theta \theta }}{{r}^{2}})-\frac{8A\nu }{k}\frac{r{z}_{,r}+{z}_{,\theta \theta }}{{r}^{4}}\simeq 0,\end{array}$$

subjected to

83 $$z=0,\,{\nabla }_{p}z=0\,{\rm{and}}\,{z}_{,rrr}+\frac{{z}_{,rr}}{r}-\frac{{z}_{,r}}{{r}^{2}}-\frac{2{z}_{,\theta \theta }}{{r}^{3}}+\frac{{z}_{,\theta \theta r}}{{r}^{2}}=\frac{2\sigma }{k},$$

where ${\mu }^{2}=\frac{2\lambda }{k}$. A complete analytical solution of the above PDEs is available via the method presented in^33–36^ and is obtained by

84 $${z}_{1}(r,\theta )={z}_{11}(r,\theta )+{z}_{12}(r,\theta ),$$

85 $$\begin{array}{rcl}{z}_{11} & = & \sum _{m=0}^{\infty }\,{K}_{n}(\frac{\mu }{\sqrt{2}}r)\,[{A}_{m}+{B}_{m}\theta +{C}_{m}\,\sin \,m\theta +{D}_{m}\,\cos \,m\theta ]\\  &  & +\,{K}_{n}(\frac{\mu }{\sqrt{2}}r)\,[{A}_{0}+{B}_{0}\theta +{D}_{0}]\\  &  & +\,\sum _{m=1}^{\infty }\,{K}_{n}(\frac{\mu }{\sqrt{2}}r)[{A}_{m}+{B}_{m}\theta +{C}_{m}\,\sin \,m\theta +{D}_{m}\,\cos \,m\theta ],\end{array}$$

and

86 $$\begin{array}{rcl}{z}_{12} & = & \sum _{m=0}^{\infty }[{C}_{m}{I}_{q}(\frac{\mu }{\sqrt{2}}r)+{D}_{m}{K}_{q}(\frac{\mu }{\sqrt{2}}r)\\  &  & +\,{E}_{m}{J}_{q}(\frac{\mu }{\sqrt{2}}r)+{F}_{m}{Y}_{q}(\frac{\mu }{\sqrt{2}}r)]\,[{A}_{m}\,\sin \,m\theta +{B}_{m}\,\cos \,m\theta ]\\  &  & +\,{r}^{\sqrt{\frac{{\mu }^{2}}{2}}}[{C}_{0}{I}_{0}(\frac{\mu }{\sqrt{2}}r)+{D}_{0}{K}_{0}(\frac{\mu }{\sqrt{2}}r)\\  &  & +{E}_{0}\,{J}_{0}(\frac{\mu }{\sqrt{2}}r)+{F}_{0}{Y}_{0}(\frac{\mu }{\sqrt{2}}r)]\,[{B}_{0}]\\  &  & +\,\sum _{m=0}^{\infty }\,{r}^{\sqrt{\frac{{\mu }^{2}}{2}}}[{C}_{m}{I}_{q}(\frac{\mu }{\sqrt{2}}r)+{D}_{m}{K}_{q}(\frac{\mu }{\sqrt{2}}r)\\  &  & +{E}_{m}{J}_{q}(\frac{\mu }{\sqrt{2}}r)+{F}_{m}{Y}_{q}(\frac{\mu }{\sqrt{2}}r)]\,[{A}_{m}\,\sin \,m\theta +{B}_{m}\,\cos \,m\theta ],\end{array}$$

where ${n}^{2}=\frac{4A\nu }{k}+\frac{{m}^{2}}{2}$ and ${q}^{4}=\frac{8{m}^{2}A\nu }{k}$. The solutions are the first and second kinds of the modified Bessel functions and usual Bessel functions of the order *n* and *q* with parameter *μ*, denoted conventionally by, *I*_*n*_, *K*_*n*_, *J*_*n*_, and *Y*_*n*_, respectively^37^. Similarly as in the rectangular case, it is found that the intra-membrane viscosity, in the case of circumferentially dominant flow, leads to straining effects on the membrane (Fig. 3) and the resulting transverse deflection is reduced with increasing velocity of viscous flow (Fig. 4a). More importantly, the viscous flow gives rise to wrinkling phenomena when $\frac{A\nu }{\lambda }\ge {10}^{-9}$. Based on our analysis of a rectangular portion of membranes and the observations from^9^ where no interactions are considered, we infer that the interaction forces between the membrane and the substrate give rise to wrinkling phenomena. Further clarification of such phenomena is, however, beyond the scope of present study due to the paucity of available data. It is shown in Fig. 4(a) that both the amplitude of wrinkles and the magnitude of transverse deflections decay away as they approach to far field boundary. Also, Fig. 4(b) illustrates that the solutions from the proposed model (dot lines) reduce to those predicted by the existing model (solid lines)^15^ in the limit of vanishing viscous flow (i.e. $A\simeq 0$). In other words, the presented solution is general enough to accommodate the particular solution where the viscous effects are not integrated.

### Example 2: Radially dominant flow

In this section, radially dominant flow is considered where we have

87 $${v}_{\theta }=-\,A\theta \,{\rm{and}}\,{v}_{r}=A.$$

Similar to the previous case, Eqs (77 and 78), together with the above yield

88 $$\begin{array}{l}({z}_{rrrr}+\frac{2{z}_{rrr}}{r}+\frac{{z}_{rr}}{{r}^{2}}+\frac{2{z}_{rr\theta \theta }}{{r}^{2}}+\frac{2{z}_{r\theta \theta }}{{r}^{3}}+\frac{{z}_{\theta \theta \theta \theta }}{{r}^{4}})\\ \,-\,{\mu }^{2}({z}_{rr}+\frac{{z}_{r}}{r}+\frac{{z}_{\theta \theta }}{{r}^{2}})-\frac{8A\nu }{k}{z}_{rr}=0.\end{array}$$

**Figure 5:**
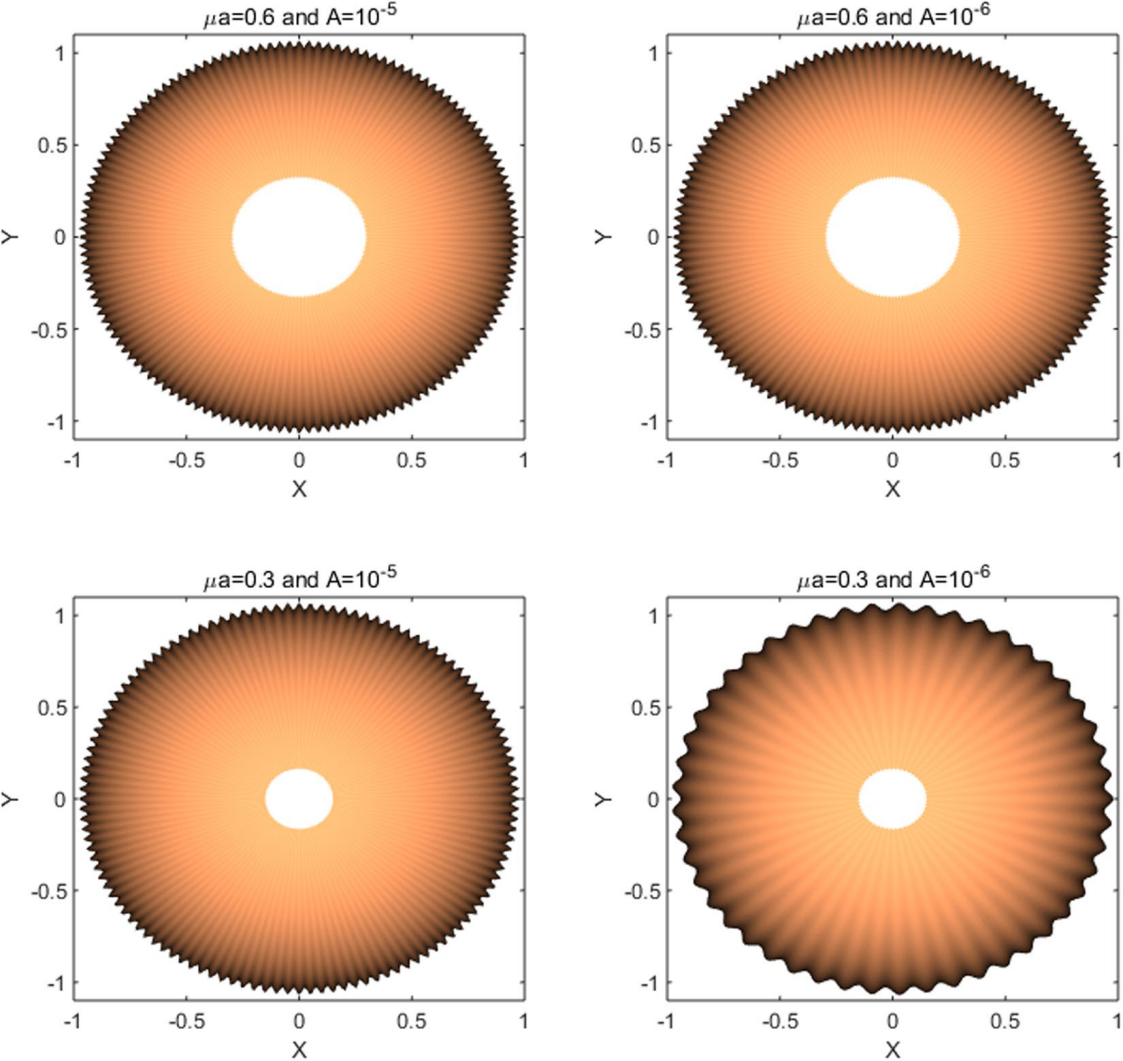
Number of wrinkles on lipid membranes with respect to *A* and *μa* (inner radius).

**Figure 6:**
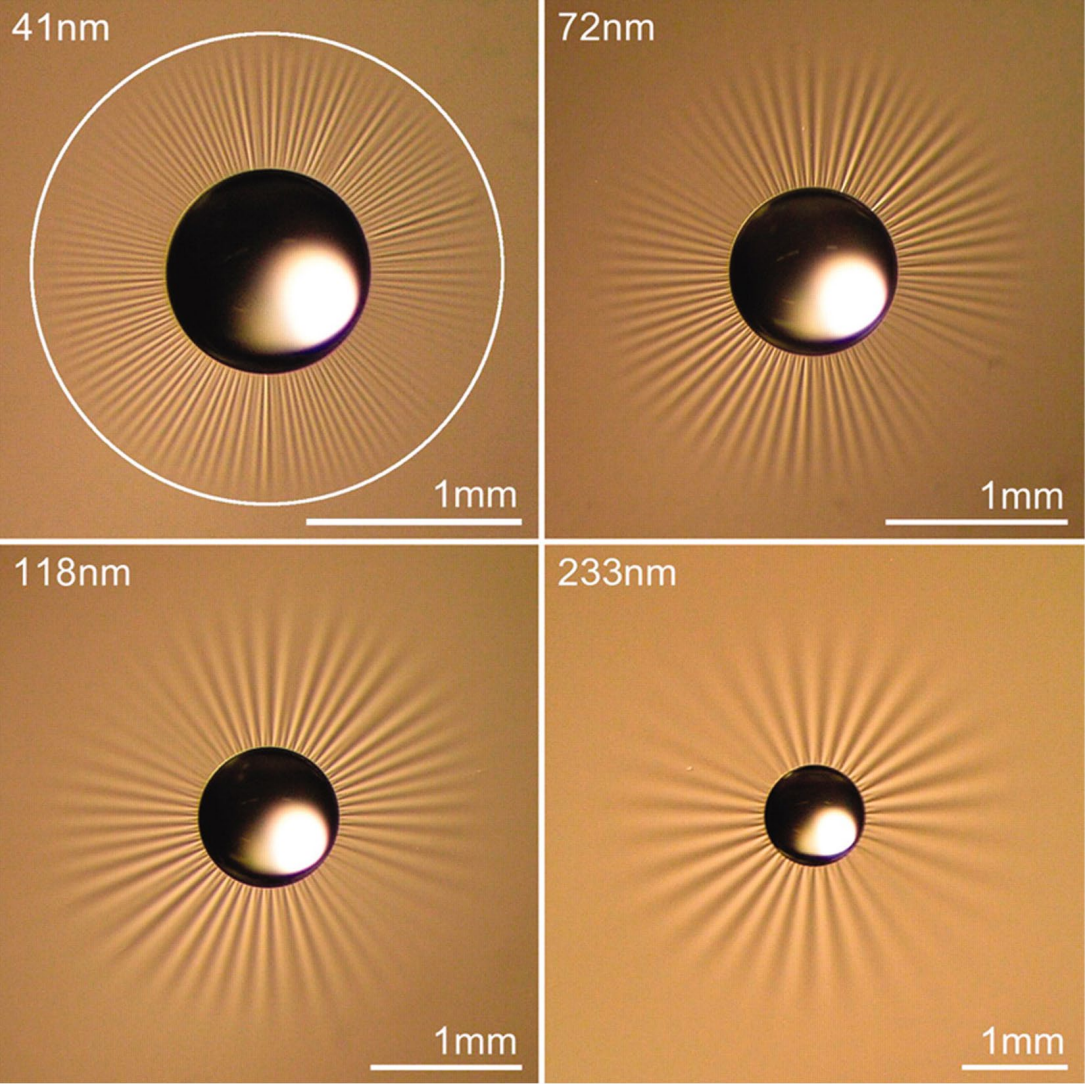
Number of wrinkles on thin polymer films^10^. From [Huang, J. *et al*. 50–653 (2007)]. Reprinted with permission from AAAS.

**Figure 7:**
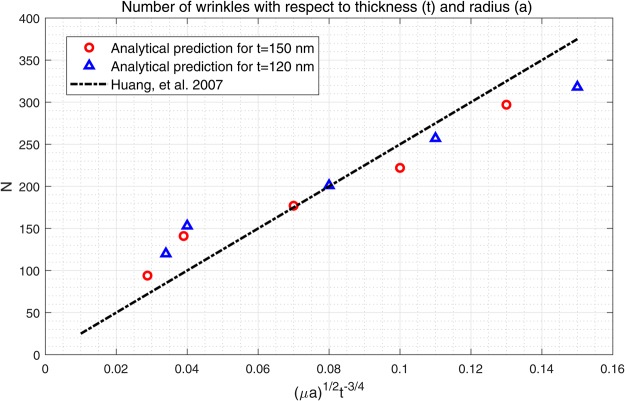
Comparison with the experimental result in^10^.

Again, using the methods in^33–36^, the solution of Eq. (88) is obtained by the following explicit form;

89 $${z}_{2}(r,\theta )={z}_{21}(r,\theta )+{z}_{22}(r,\theta ),$$

90 $$\begin{array}{rcl}{z}_{21} & = & \sum _{m=0}^{\infty }\,{K}_{s}(\frac{\mu }{\sqrt{2}}r)\,[{A}_{m}+{B}_{m}\theta +{C}_{m}\,\sin \,m\theta +{D}_{m}\,\cos \,m\theta ]\\  &  & +\,{K}_{0}(\frac{\mu }{\sqrt{2}}r)[{A}_{0}+{B}_{0}\theta +{D}_{0}]\\  &  & +\,\sum _{m=1}^{\infty }\,{K}_{s}(\frac{\mu }{\sqrt{2}}r)[{A}_{m}+{B}_{m}\theta +{C}_{m}\,\sin \,m\theta +{D}_{m}\,\cos \,m\theta ],\end{array}$$

and

91 $$\begin{array}{rcl}{z}_{22} & = & \sum _{m=0}^{\infty }\,{r}^{\sqrt{\frac{{\mu }^{2}}{2}}}[{C}_{m}{I}_{t}(\frac{\mu }{\sqrt{2}}r)+{D}_{m}{K}_{t}(\frac{\mu }{\sqrt{2}}r)+{E}_{m}{J}_{t}(\frac{\mu }{\sqrt{2}}r)\\  &  & +\,{F}_{m}{Y}_{t}(\frac{\mu }{\sqrt{2}}r)]\,[{A}_{m}\,\sin \,m\theta +{B}_{m}\,\cos \,m\theta ]\\  &  & +\,{r}^{\sqrt{\frac{{\mu }^{2}}{2}}}[{C}_{0}{I}_{0}(\frac{\mu }{\sqrt{2}}r)+{D}_{0}{K}_{0}(\frac{\mu }{\sqrt{2}}r)\\  &  & +\,{E}_{0}\,{J}_{0}(\frac{\mu }{\sqrt{2}}r)+{F}_{0}{Y}_{0}(\frac{\mu }{\sqrt{2}}r)]\,[{B}_{0}]\\  &  & \sum _{m=0}^{\infty }\,[{C}_{m}{I}_{t}(\frac{\mu }{\sqrt{2}}r)+{D}_{m}{K}_{t}(\frac{\mu }{\sqrt{2}}r)+{E}_{m}{J}_{t}(\frac{\mu }{\sqrt{2}}r)\\  &  & +\,{F}_{m}{Y}_{t}(\frac{\mu }{\sqrt{2}}r)]\,[{A}_{m}\,\sin \,m\theta +{B}_{m}\,\cos \,m\theta ],\end{array}$$

where and ${s}^{2}=\frac{{m}^{2}}{2}$ and *t*^2^ = *mμ*. Similar to the circular case, radially arranged wrinkles begin to form as the parameter exceeds the critical value of $\frac{A\nu }{\lambda }\ge 3\times {10}^{-10}$ and they vanish at the remote boundary. The number of radial wrinkles increases with the growing effects of viscous flow (Fig. 5) and with larger radius of an inner circle. A similar tendency can be found in the work of^10^ (See, Fig. 6) where the authors measure the number of wrinkles on thin polymer films under the compatible settings as considered in the present work. In addition, we assimilate the experiments in^10^ by using the proposed analytical model and present the results in Fig. 7. Although, the obtained model is not intended for the analysis of thin polymer films, it still provides reasonable agreement with the results in^10^ (see, Fig. 7) that the number of wrinkles is sensitive to both the thickness ‘*t*’ (inversely proportional) and the inner radius ‘*a*’ (proportional). The results are also align with the theoretical developments regarding to finely wrinkled states of the membrane via the minimization of the strain-energy function and by its quasi-convexification^11,12^. Potential applications to biomechanics may include monitoring vesicle thickness and enhancing vesicle fusion processes. However, prior to these applications, it would be necessary to further clarify and/or justify the obtained results by employing the aforementioned theory.

### Data acquisition

Figures in the manuscript are prepared by visualizing analytical solutions presented in the manuscript. For the purpose, a commercial software (MATLAB) is used.

### Ethics statement

This work did not involve any collection of human data.

## Data Availability

This work does not have any experimental data.
